# Low Goiter Rate Associated with Small Average Thyroid Volume in Schoolchildren after the Elimination of Iodine Deficiency Disorders

**DOI:** 10.1371/journal.pone.0141552

**Published:** 2015-10-29

**Authors:** Peihua Wang, Hong Sun, Li Shang, Qinglan Zhang, Yingxia He, Zhigao Chen, Yonglin Zhou, Jingjing Zhang, Qingqing Wang, Jinkou Zhao, Hongbing Shen

**Affiliations:** 1 Department of Epidemiology and Statistics, School of Public Health, Nanjing Medical University, Nanjing, China; 2 Department of Environmental and Endemic Disease Control and Prevention, Jiangsu Provincial Center for Disease Control and Prevention, Nanjing, China; National Institute for Viral Disease Control and Prevention, CDC, China, CHINA

## Abstract

**Background:**

After the implementation of the universal salt iodization (USI) program in 1996, seven cross-sectional school-based surveys have been conducted to monitor iodine deficiency disorders (IDD) among children in eastern China.

**Objectives:**

This study aimed to examine the correlation of total goiter rate (TGR) with average thyroid volume (Tvol) and urinary iodine concentration (UIC) in Jiangsu province after IDD elimination.

**Design:**

Probability-proportional-to-size sampling was applied to select 1,200 children aged 8–10 years old in 30 clusters for each survey in 1995, 1997, 1999, 2001, 2002, 2005, 2009 and 2011. We measured Tvol using ultrasonography in 8,314 children and measured UIC (4,767 subjects) and salt iodine (10,184 samples) using methods recommended by the World Health Organization. Tvol was used to calculate TGR based on the reference criteria specified for sex and body surface area (BSA).

**Results:**

TGR decreased from 55.2% in 1997 to 1.0% in 2009, and geometric means of Tvol decreased from 3.63 mL to 1.33 mL, along with the UIC increasing from 83 μg/L in 1995 to 407 μg/L in 1999, then decreasing to 243 μg/L in 2005, and then increasing to 345 μg/L in 2011. In the low goiter population (TGR < 3.9%), TGR was positively associated with average Tvol (r = 0.99); UIC showed a non-linear association with average Tvol, and UIC > 300 μg/L was associated with a smaller average Tvol in children.

**Conclusions:**

After IDD elimination in Jiangsu province in 2001, lower TGR was associated with smaller average Tvol. Average Tvol was more sensitive than TGR in detecting the fluctuation of UIC. A UIC of 300 μg/L may be defined as a critical value for population level iodine status monitoring.

## Introduction

Universal salt iodization (USI), defined as the fortification of all salt used for human and animal consumption with iodine, is the main strategy recommended by the World Health Organization (WHO) to control iodine deficiency disorders (IDD) worldwide [1]. Since 1996, USI has been implemented in Jiangsu province, which is located in eastern China and has a population of approximately 79 million people. We conducted 8 cross-sectional school-based surveys from 1995 to 2011 to monitor salt iodine and IDD in the province, with one study undertaken in 1995 prior to the implementation of USI. Thyroid size is the most commonly used parameter in baseline assessments of IDD severity and plays a role in assessing the long-term impact of control programs [1]. In addition to palpation, thyroid size can also be determined using ultrasonography, which provides quantitative measurement of thyroid volume (Tvol) and is currently considered the most reliable tool for measuring Tvol [2, 3]. Since the introduction of USI, the total goiter rate (TGR) measured by ultrasonography decreased significantly in Jiangsu, and a very low TGR has been maintained in recent years. The World Health Organization (WHO) defines the IDD as a public health problem based on a TGR of 5% or higher in schoolchildren aged 6 to 12 years old but does not suggest an appropriate lower limit of TGR as the criterion of IDD elimination. We knew exactly that TGR would decrease with average Tvol in populations suffering from IDD [4], but there might be no necessary correlation between average Tvol and TGR. In a population with naturally enough but not more than adequate iodine intake, the average thyroid volume is supposed to be stable, but goitre can still exist, because goitre might be caused by many other factors except iodine deficiency, such as Graves’ disease. And if TGR in this population changes because of the variable incidence of Graves’ disease (supposing that the changes of thyroid volume in this people with goitre are not significant enough to change the average thyroid volume of the whole population), then the TGR will have no association with the average thyroid volume. But would the very low TGR still be positively associated with further small average Tvol in populations after the elimination of IDD? There’s no previous studies could answer this hypothesis.

TGR and Tvol vary with iodine intakes among populations [4, 5]. The WHO defines excessive iodine intake using a median urinary iodine concentration (UIC) equal to or greater than 300 μg/L and more than adequate iodine intake as UIC ≥ 200 μg/L. Similar to IDD, excessive iodine intake has also received substantial attention [6]. Extremely high iodine intake associated with endemic goiter in children has been previously described in coastal Japan, where daily iodine intake from seaweed is > 10,000 μg/d [7]. In Chinese children, the reason for high iodine intake is generally attributed to iodine-rich drinking water, which is also associated with increased serum thyrotropin and Tvol [8, 9]. UIC ≥ 500 μg/L has also been found to be associated with abnormally large Tvol [10]. In populations that have a UIC between 300 and 500 μg/L, few studies have examined the relationship between average Tvol and iodine intake. Either no association has been found [8] or small Tvol might be associated with excessive iodine intake [11, 12].

This study aimed to examine the correlation of TGR with average Tvol and iodine intake in Jiangsu province where USI has been implemented for 16 years.

## Materials and Methods

### Ethics

The survey was developed by Jiangsu Provincial Center for Disease Control and Prevention (Jiangsu CDC) and approved by its Ethics Committee. The surveys were performed according to the guidelines laid down in the Declaration of Helsinki. Written informed consents were obtained from school teachers and guardians of each participating student. The Jiangsu CDC conducted all surveys, including field data collection and laboratory measures, with logistic support from local CDCs in selected areas.

### Subjects and sampling

According to the WHO’s guideline for monitoring IDD in very large populations [1], we selected 8- to 10-year-old school children as survey subjects. The surveys were conducted from March to April in 1995, 1997, 1999, 2001, 2002, 2005, 2009 and 2011. A probability proportional-to-size (PPS) cluster sampling was applied in the surveys. All 100 rural counties or urban districts in Jiangsu province without known excessively high iodine concentrations in the drinking water were listed with respective total population size. From the list, 30 counties or districts were selected following a PPS approach [1]. The list of schools was then acquired from the selected counties or districts. A random number table was used to select one school from each of the 30 counties or districts. In the selected 30 schools, the student registers were acquired, and 40 schoolchildren aged 8 to 10 years old were chosen at random (20 males and 20 females). Children absent from school on the day of the survey and those with any known chronic disease were not included.

The Tvol of each subject was measured except in 1995. Household salt samples were collected from the houses of the selected children. Casual spot urinary samples of at least 12 students randomly selected from a school (40 children) were collected, with at least two males and two females selected from each age group (8, 9, 10 year olds). Samples of urine and salt were separately collected by different trained personnel to prevent iodine contamination between salt samples and urine samples.

### Laboratory measures of iodine concentration in salt and urine samples

The iodine concentration in the salt samples was determined by iodometric titration with sodium thiosulfate using starch as an external indicator [13], according to China Technical Standard GB/T 13025.7–1999 [14]. The adequacy of iodine concentration in salt was defined as 20–60 mg/kg prior to 2000 and as 20–50 mg/kg afterwards.

The iodine concentration in urine (UIC) samples were measured based on the Sandell-Kolthoff reaction (WS/T 107–2006) [15]. Aliquots of urine (250 μL) were prepared with ammonium persulfate at 100°C. Arsenious acid and ceric ammonium sulfate were then added. The decrease in yellow color over a fixed time was detected with a spectrometer, and a standard curve was plotted with a known amount of iodine [16]. All laboratory analyses were performed in the iodine laboratory of the Jiangsu CDC, which is accredited by the National Iodine-deficiency Disorders Reference Laboratory in Beijing. The detection limit for UIC was 3 μg/L, and our intra-assay coefficient of variation (CVs) were 4.6% at UIC = 76.2 ± 3.5 μg/L and 3.3% at UIC = 223.9 ± 7.5 μg/L. The intra-assay CVs for salt iodine detection were 1.0% at 31.7 ± 0.3 mg/kg and 1.9% at 16.2 ± 0.3 mg/kg. Individual UIC varied each day and within a given day [17, 18], so it could not be analyzed as a reliable independent variable. However, the variation of UIC tended to even out among populations, and Bourdoux had noted that a "sufficient" number of spot urine samples should be 50–100 [19]. In the present study, more than 400 samples of spot urine were measured to provide a median UIC.

### Determination of thyroid volume

Tvol was determined by real-time echography with Medison mycolor 202 (Medison Tower, 997–10 Daechi-dong, Gangnam-gu, Seoul, Korea) using a 7.5 MHz linear array transducer. Two technicians (QZ and LS), trained by the National Institute of Iodine Deficiency Disorders in Harbin, performed all the measurements. Longitudinal and transverse scans were taken, allowing measurement of the depth, width, and length of each lobe. The volume of the lobe was calculated using the following formula: volume (mL) = 0.479 × depth (cm) × width (cm) × length diameter (cm). The lobe volumes were then summed; the volume of the isthmus was not included. The intra-observer variabilities, evaluated by the different values of our two trained technicians performing repeated measurements to 4% of the samples, were -0.064 to 0.155 and -0.115 to 0.158. The inter-observer variability evaluated by duplicate measurements of approximately 4% of samples was -0.178 to 0.201. The weight and height of each child wearing light clothing without shoes or hats were measured. Body surface area (BSA) was calculated using the following formula: BSA = weight (kg)^0.425^ × height (cm)^0.725^ × 71.84 × 10^−4^.

### Statistics

Median UIC was calculated for each survey as the iodine intake value of each survey. Subjects with missing UIC data were not included in the statistical analysis of UIC and its association with average Tvol. According to the WHO guideline, the term “goiter” refers to a thyroid gland that is enlarged, so goiter was defined using sex- and BSA-specific reference criteria for Tvol [20]. TGR was calculated by dividing the abnormally large thyroid volume (goiter) by the total examined. TGR across surveys was compared using a Chi square test. To normalize the distributions, Tvol was log transformed, and the geometric means of Tvol were calculated. Pearson correlation and linear regression were performed to find the correlation between geometric mean of Tvol and TGR in each survey. The difference of Tvol across surveys was compared using analysis of covariance (ANCOVA), with age, BSA, and sex as covariates. BSA-adjusted Tvol was achieved by dividing Tvol by the corresponding BSA. Graphpad prism 6 (GraphPad Software, Inc. CA) was used to draw figures, and R software (version 3.03 for Windows) was used for statistical analysis.

## Results

### Iodine concentrations in household salt

The average iodine concentration in salt was higher than 40 mg/kg in the 1990s and then decreased to less than 30 mg/kg after 2002 (**Table 1**) (data from 1995 is not shown in Table 1 because USI had not been implemented at that time). The use of iodized salt in household increased steadily, and this level continued at approximately 98% after 2001. The percentage of salt with adequate iodine used in household has been greater than 94% since 2001. Both values are greater than the WHO’s criteria on IDD elimination [1].

**Table 1:** Status of iodized salt used in household in Jiangsu province from 1997 to 2011.

Year	N	Mean^a^ (mg/kg)	Std (mg/kg)	CV (%)	Range (mg/kg)	Use of iodized salt in household (%)	Use of salt with adequate iodine ^b^(%)
1997	1194	44.1	27.0	61.1	0–240.1	90.8	65.5
1999	1190	46.2	24.5	53.1	0–281.0	89.0	65.7
2001	1160	35.8	13.5	37.6	2.1–82.0	94.4	77.3
2002	3075^c^	32.0	7.4	23.2	0–71.3	97.9	96.2
2005	1165	29.9	7.1	23.7	0–62.8	98.2	94.2
2009	1200	29.0	7.9	27.2	0–98.7	97.5	94.5
2011	1200	29.6	5.8	19.6	0–50.0	98.8	97.2

a. Mean of the iodine content in salt used in household

b. Use of salt with adequate iodine was defined as 20–60 mg/kg before year 2000 and 20–50 mg/kg after 2000.

c. The salts were also sampled in other places in addition to the 30 countries.

### Thyroid volume and total goiter rate

**Fig 1:**
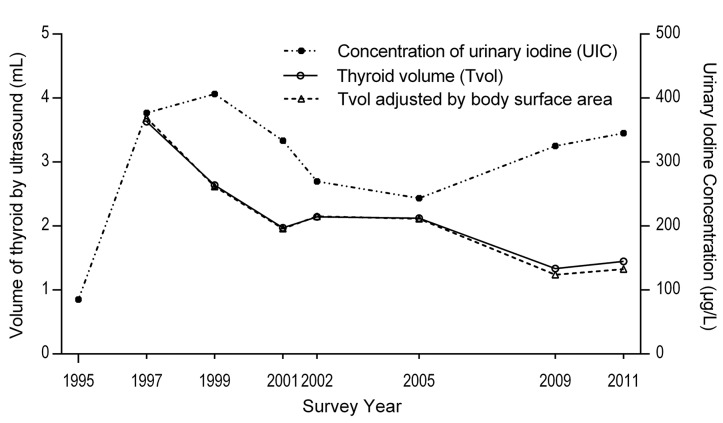
The geometric mean of thyroid volume (Tvol) and median urinary iodine concentration (right y-axis) in the surveys. Body surface area (BSA) was used to adjust Tvol and slightly affected the geometric mean of Tvol.

We measured Tvol in 8,314 children (**S1 Table**). The geometric mean value of Tvol with or without BSA-adjustment showed no significant difference in each survey (P = 0.28, **Fig 1**). The geometric mean of Tvol decreased from 3.63 mL in 1997 to 1.97 mL in 2001 and then maintained at approximately 2.10 mL from 2002 to 2005, followed by a remarkable decrease in 2009 to 1.33 mL. Generally, average Tvol decreased over time, with a slight increase observed between 2001 and 2002 and between 2009 and 2011 (Fig 1). Geometric means of Tvol by ages, gender and surveys was summarized in **Table 2,** and the median values for Tvol measured by ultrasound according to gender and age from Zimmermann [20] were also listed. Tvol increased with age (P<0.01) across the surveys, and the girls had significantly higher values for Tvol than did the boys (P<0.01).More importantly, in the surveys after 2000, the average Tvol of our children were significantly smaller than those reported by Zimmermann (P<0.01).

**Table 2:** Thyroid volume in children by ages and gender in each survey.

	8 years old, GM (95% CI)		9 years old, GM (95% CI)		10 years old, GM (95% CI)	
Survey years	Girls	Boys	Girls	Boys	Girls	Boys
1997	3.22(3.06,3.39)	3.31(3.14,3.48)	3.46(3.33,3.60)	3.50(3.36,3.65)	4.09(3.94,4.25)	3.78(3.64,3.93)
1999	2.39(2.27,2.53)	2.40(2.29,2.52)	2.61(2.47,2.76)	2.69(2.54,2.84)	3.00(2.84,3.16)	2.79(2.63,2.95)
2001	1.97(1.88,2.05)	1.90(1.83,1.97)	2.00(1.91,2.10)	1.97(1.90,2.05)	2.01(1.91,2.11)	1.98(1.90,2.06)
2002	2.05(1.97,2.13)	2.05(1.97,2.13)	2.10(2.02,2.17)	2.07(1.99,2.14)	2.28(2.20,2.36)	2.25(2.18,2.33)
2005	1.98(1.90,2.06)	1.95(1.85,2.04)	2.12(2.03,2.21)	2.11(2.01,2.20)	2.29(2.18,2.41)	2.26(2.17,2.36)
2009	1.25(1.18,1.33)	1.23(1.17,1.31)	1.33(1.26,1.40)	1.31(1.24,1.38)	1.46(1.38,1.55)	1.40(1.34,1.47)
2011	1.33(1.24,1.41)	1.40(1.32,1.48)	1.46(1.36,1.56)	1.44(1.35,1.54)	1.53(1.43,1.64)	1.51(1.41,1.62)
Ref ^a^	2.08	2.03	2.40	2.30	2.76	2.59

a. Median values for Tvol according to gender and age by MB Zimmermann, 2004 (20).

**Fig 2:**
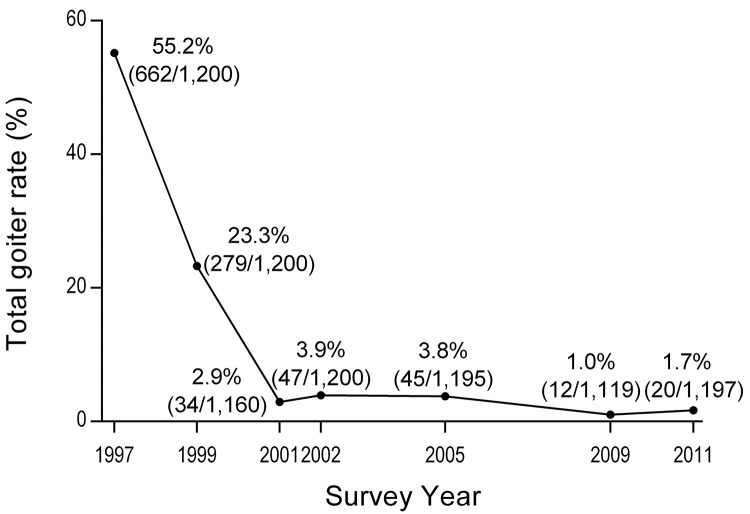
Thyroid goiter rate (TGR) among children aged 8 to 10 in Jiangsu province from 1997 to 2011 based on the WHO sex- and BSA-specific reference criteria of 2007.

TGR significantly decreased from 1997 to 2011 (P < 0.001) **(Fig 2)**, with a substantial reduction from 1995 to 2001 (P < 0.001). There was no significant difference between 2001 and 2002 (P = 0.11) or 2009 and 2011 (P = 0.15), but there was a significant reduction from 2005 to 2009 (P < 0.001).

**Fig 3:**
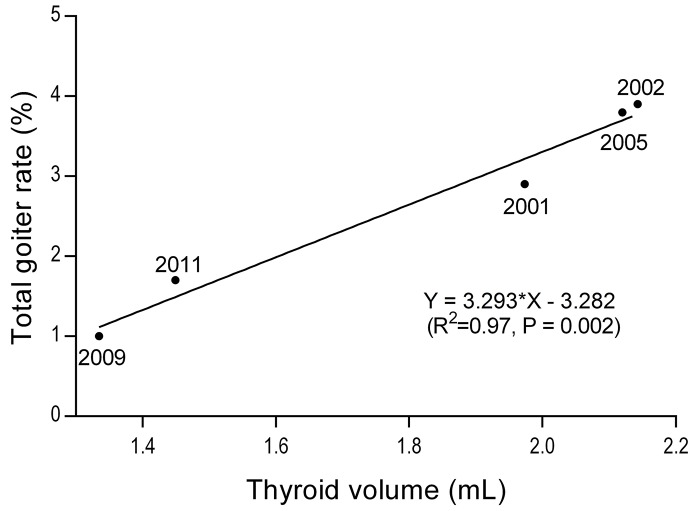
Correlation between average thyroid volume (Tvol) and thyroid goiter rate (TGR) after elimination of IDD. The numbers around points indicate the survey years. The data of TGRs and geometric means of Tvol from 2001 to 2011 were analyzed.

**Fig 3** shows a close correlation of average Tvol with TGR (Pearson’s r = 0.99, P = 0.002) from 2001 to 2011 after IDD was eliminated.

### Urinary iodine concentration

**Fig 4:**
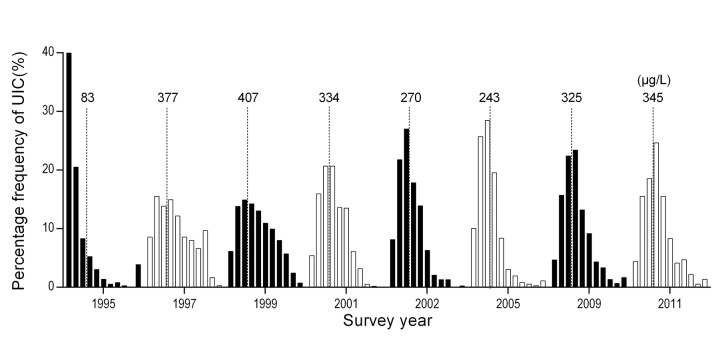
Distribution of concentration of urinary iodine (UIC) in the surveys. The UIC in each survey was categorized into 11 groups by UIC <100 μg/L, < 200 μg/L, etc. to < 1,000 μg/L and ≥1,000 μg/L. Each bar represents the percentage frequency of one group. The dashed lines show the position of UIC = 300 μg/L. The number above each bar is the median UIC in each survey (unit: μg/L). This Fig shows that the distribution of UIC was concentrated around the median value, and few proportions with either very low or very high values were observed after 2000.

We measured 4,767 urine samples for UIC. There were a total of 17 missing UIC data across eight surveys (17/4,784), which leaked during transport. As shown in Fig 1, in the first 5 years of the USI program, the median UIC was at the highest concentration and reached 406 μg/L in 1999. After the decrease in iodine concentration in salt (Table 1), the median UIC continuously decreased to 334 μg/L in 2001 and then decreased to 270 μg/L in 2002. It further decreased to 243 μg/L in 2005. From 2005 to 2011, a sharp increase in UIC was observed, with a median value of 325 μg/L in 2009 and 345 μg/L in 2011. The distribution of UIC was concentrated around the median value, and small proportions with either very low or very high values were observed after year 2001, as shown in **Fig 4.**

### Tvol of various levels of UIC

We observed a non-linear change of average Tvol with UIC (Fig 1), and UIC = 300 μg/L was strongly suggested as a boundary value for this changing pattern of Tvol. When UIC decreased from 334 μg/L in 2001 to 270 μg/L in 2002 (Fig 1), a significant increase of average Tvol was observed from 1.97 mL to 2.14 mL (P < 0.01, ANCOVA). When UIC increased from 243 μg/L in 2005 to 325 μg/L in 2009, another significant decrease of average Tvol from 2.12 mL to 1.33 mL (P < 0.01 in ANCOVA) occurred. When UIC remained less than 300 μg/L during 2002 to 2005, average Tvol remained stable at 2.14 mL in 2002 to 2.12 mL in 2005, coinciding with UIC decreasing from 270 μg/L to 243 μg/L. When UIC was higher than 300 μg/L from 2009 to 2011, average Tvol significantly increased from 1.33 mL to 1.45 mL (P < 0.01). We classified average Tvol in which TGR was less than 3.9% into two groups (median UIC < 300 μg/L (in 2002 and 2005) and > 300 μg/L (in 2001, 2009 and 2011)), and we found average Tvol was significantly small if UIC > 300 μg/L (P < 0.01).

## Discussion

This study is the first to reveal a positive correlation between average Tvol and TGR in a population after the elimination of IDD. Zimmermann [4] showed decreased thyroid size and lower goiter prevalence after introduction of iodized salt in the first 5 years of USI, which might indicate the goiter’s response to increased iodine intake. Our study extended the observation period to 16 years after USI and showed that TGR decreased to normal. Additionally, we found that TGR was still positively correlated with average Tvol. If IDD is monitored as a public health problem, TGR becomes less sensitive after IDD has been eliminated. However, how small the Tvol should be in this era of IDD elimination remains unknown. Little is known about the possible adverse effects of smaller thyroid volume. Given the lag period between changes in UIC and changes in Tvol, it is important to maintain UIC within a particular range to maintain low average Tvol; for example in 2009, we found the low Tvol of 1.33 mL, which corresponds to the TGR of 1% in 2009 (8 years after IDD elimination).

We also observed that the fluctuations in average Tvol were associated with UIC if TGR < 3.9% and IDD was eliminated. Tvol with UIC showed a three-stage relationship, and a UIC of 300 μg/L was observed to be an important cut-off point. First, if UIC was less than 300 μg/L (in 2002 and 2005), UIC showed no effects on average Tvol. The result was supported by Zou [3], who found that UIC in this concentration range would not affect Tvol. Second, if UIC increased from less than 300 μg/L to greater than 300 μg/L, a significant decrease in average Tvol was observed. This effect might be reversible because the average Tvol increased as UIC decreased from 2001 to 2002. This result mainly suggested that average Tvol continued decreasing with the increase in UIC at approximately 300 μg/L after the elimination of IDD. This result is consistent with previous studies which state that the Tvol of 8-year-old boys with normal iodine excretion is greater than that of those with relative high iodine excretion, as shown in a Japanese study [21] and a study conducted in Brazil [11]. Third, if UIC > 300 μg/L, Tvol began increasing with UIC (Fig 1), but the TGR between 2009 and 2011 showed no significant difference (P = 0.15). UIC > 300 μg/L is defined as excessive iodine intake by the WHO. Excessive iodine intake, which generally occurred in areas where food and drinking water are rich in iodine, has been associated with goiters in children [5, 22]. However, different cut-off points of UIC have been reported in examining the association with goiter rate. UIC > 900 μg/L is associated with a TGR of > 10% in northwest Jiangsu in China, where water iodine exceeds 300 μg/L [23]. Among 6- to 12 year-old children, UIC ≥500 μg/L is associated with an increase in thyroid size as measured by ultrasonography [10], based on the data from coastal Hokkaido (median UIC was 728 μg/L). In other sites with a high prevalence of UIC > 500 μg/L, no significant increase in Tvol at higher UIC was observed [10]. In the present study, we observed a significant increase in average Tvol in the children with UIC > 300 μg/L from 2009 to 2011; however, with much smaller average Tvol than if UIC < 300 μg/L (Fig 1). This finding suggests that a UIC of 300 μg/L in school children may be defined as a critical value.

The salt iodine concentration in Jiangsu peaked before 2000. However, no similar exposure was observed in Shenzhen, where the median UIC was 207.1 μg/L in 2011 and 232.2 μg/L in 2009 [24], or in Zhejiang province, where the median UIC was only 170 μg/L in 6- to 12-year-old children [3] in 2011. Therefore, salt iodine might explain why our monitored UIC levels were much higher than those in other Chinese districts [24]. However, salt iodine might not be the only factor because the status of iodized salt has been stable since 2001 but UIC has been variable (Fig 1). Our monitoring of water iodine showed that in the 30 surveyed counties, the median concentration of water iodine also remained stable and decreased during our surveys: 17.3 μg/L in 1997, 12.6 μg/L in 2002, and 12.4 μg/L in 2011. The increase in UIC might also be attributed to other factors, including improved nutrition intake in children, such as the consumption of more seaweed [7] and iodine-rich meat and milk [24].

Our results also showed the solid progress toward the implementation of USI in Jiangsu province. Great achievements in USI have decreased the goiter rate from 55.2% to 1%, with the use rate of adequate iodized salt reaching and maintaining approximately 95% (Table 1). The achievement of USI in China has also been verified in Shenzhen special district of China [24], Zhejiang province [3], Shanghai [25] and Jiangsu [26]. Our results indicate that IDD has been eliminated in Jiangsu province since 2001.

The findings of this study are subject to several limitations. First, we used a cross-sectional design, and the subjects belonged to different birth cohorts, which might cause unquantifiable bias. Second, because we were limited by the relatively high UIC observed throughout the surveys, we could not observe the correlation of average Tvol with UIC < 243 μg/L. In a survey conducted in Shenzhen of China, decreasing TGR was observed when the median UIC dropped from 278.8 μg/L to 207.1 μg/L, which suggested that a possible association between average Tvol and UIC < 300 μg/L might still exist. Third, the association between Tvol and UIC is based on two surveys and may possibly be affected by the lag period. Subsequent surveys will provide additional observations. However, this report is the first study examining the relationship among UIC, average Tvol and TGR with periodic surveys during 16 years after USI.

## Conclusion

IDD has been eliminated by USI in Jiangsu province since 2001. As IDD has been persistently eliminated as evidenced by UIC, TGR and use of salt iodization, lower TGR is associated with smaller average Tvol, and average Tvol is a more sensitive indicator than TGR to the fluctuation of UIC. Our findings suggest a UIC of 300 μg/L may be held to be a critical value at the population level for iodine status monitoring and program evaluation. Iodine concentration in salt should be evaluated periodically to maintain UIC in the optimal range in the context of overall socioeconomic development.

## Supporting Information

S1 Table The original data from eight surveys from 1995 to 2011, including year, sex, age, body surface area (BSA), urinary iodine concentration; thyroid volume. (XLSX)
